# LncRNA-mRNA Expression Profiles of Osteoclast After Conditional Knockout HIF-1α

**DOI:** 10.3389/fgene.2022.909095

**Published:** 2022-06-21

**Authors:** Yuanye Tian, Qi Shao, Jiahong Gu, Yi Tang, Miaomiao Bie, Yangyifan Zhou, Chunan Cheng, Yi Liang, Qian Zhang, Feiwu Kang

**Affiliations:** ^1^ Department of Oral and Maxillofacial Surgery, School and Hospital of Stomatology, Tongji University, Shanghai, China; ^2^ Shanghai Engineering Research Center of Tooth Restoration and Regeneration, Shanghai, China

**Keywords:** osteoclast, lncRNA, mRNA, HIF-1α, condyle

## Abstract

**Background:** Osteoclasts, which are multinucleated cells formed by monocyte fusion, play a key role in bone resorption. Hypoxia-inducible factor (HIF)-1α is vital for the development of osteoclasts in hypoxic environments and during bone resorption. However, additional research is required to further study the HIF-1α-dependent regulation of osteoclast differentiation at the genetic level.

**Methods:** In our study, RNA sequencing (RNA-seq) was used to identify the expression profiles of long noncoding RNAs (lncRNAs) and mRNAs in conditional HIF-1α-knockout osteoclasts.

**Results:** A total of 1,320 mRNAs and 95 lncRNAs were differentially expressed. The expression of lncRNAs MSTRG.7566.12 and MSTRG.31769.2 were strongly negatively correlated with that of Mmp9, Ctsk, etc.

**Conclusion:** Our research provides a basis for further understanding the role of mRNAs and lncRNAs in conditional HIF-1α-knockout osteoclasts, and many of these molecules may be potential targets for treating bone diseases related to HIF-1α.

## 1 Introduction

Bone is a highly vascularized tissue that has a rich blood supply that maintains its nutrient and oxygen concentrations. However, bone fracture or inflammation may disrupt this circulation, leading to hypoxia of adjacent bone and marrow (Knowles, 2017). As the core molecule that senses and responds to the local oxygen environment, hypoxia-inducible factor (HIF)-1α plays a certain role in bone metabolism. Bone metabolism mainly involves bone formation and bone resorption (Weivoda et al., 2020). Osteoclasts are giant multinucleated cells derived from the fusion of bone marrow mononuclear macrophage (BMM) precursors. After fusion and maturation, BMMs differentiate into osteoclasts which play a vital role in the bone resorption process (Miron and Bosshardt, 2018). The main function of osteoclasts is to absorb bone tissue by secreting acid and collagen-degrading enzymes. Receptor activator of nuclear factor-κ B Ligand (RANKL) is a signalling factor in the differentiation of osteoclasts, and RAW264.7 cells as an alternative can be induced to form osteoclasts by RANKL (Fujisaki et al., 2007). Differentiated and mature osteoclasts secrete large amounts of substances, such as hydrogen ions and CTSK, tartrate-resistant acid phosphatase (TRAP), MMP9, and other proteins (Arnett and Orriss, 2018; Weivoda et al., 2020). HIF-1α is vital for the development of osteoclasts in hypoxic environments and during bone resorption and functions by regulating nutrient and energy sensors (Knowles, 2015). In recent decades, an increasing number of regulatory factors have been shown to be involved in the differentiation of osteoclasts, including cytokines, signalling pathway molecules, and natural compounds (Chambers, 1985; Roodman, 1999; Ono and Nakashima, 2018). Whether during osteoclast differentiation or maturation, osteoclasts are ready to respond to hypoxia. It has been reported that HIF-1α participates in osteoclast differentiation by regulating nutrient and energy sensors (Dirckx et al., 2018; Tang et al., 2019). additional research is required to further study the HIF-1α-dependent regulation of osteoclast differentiation at the genetic level.

Long non-coding RNAs (lncRNAs) are RNAs that do not encode proteins and are greater than 200 nucleotides in length (Ransohoff et al., 2018). Many non-coding genes have traditionally been considered junk DNA. With the increase in the number of gene sequencing studies, lncRNAs have been shown to play an important role in many biological activities, such as dose compensation, epigenetic regulation, cell cycle regulation and cell differentiation regulation (Ferrè et al., 2016; Quinn and Chang, 2016; Chatterjee and Sengupta, 2019), and lncRNAs have become a research hotspot in genetics. Some articles have reported the role of lncRNAs in osteoclast differentiation (Dou et al., 2016; Liu et al., 2020), but the HIF-1α-independent regulation of osteoclast differentiation at the genetic level still needs to be further investigated.

In this study, we explored the lncRNA-mRNA expression profiles associated with HIF-1α-knockout mouse osteoclast differentiation by RNA sequencing (RNA-seq) and confirmed these expression profiles by quantitative real-time polymerase chain reaction (qRT-PCR). Moreover, Gene Ontology (GO) and Kyoto Encyclopedia of Genes and Genomes (KEGG) (Kanehisa et al., 2021)analyses were used to predict the potential cellular functions of the differentially expressed mRNAs and lncRNAs. In addition, path net analysis and co-expression networks were used to predict the gene regulatory networks of osteoclast differentiation. Our study provides a new understanding of the regulation of osteoclast differentiation by lncRNAs.

## 2 Materials and Methods

### 2.1 Animals and Ethics Statement

All the mice were purchased from the Jackson Laboratory. Osteoclast-specific conditional HIF-1α-knockout mice (Cko: HIF-1αflox/flox; Ctsk cre+) were generated by intercrossing mice homozygous for a floxed HIF-1α allele with mice harbouring the Cre gene in the Cathepsin K locus (Ctsk cre+). We compared Cko mice with wild-type controls (Ctrl: HIF-1αflox/flox; Ctsk cre-) (n = 5 for each group). These methods were the same as those in our previous study (Tian et al., 2020). All methods were performed in accordance with the revised Animals (Scientific Procedures) Act 1986 in the UK and Directive 2010/63/EU in Europe. The Animal Ethics Committee of the Stomatological Hospital Affiliated to Tongji University approved all the procedures involving animals.

### 2.2 Cell Culture, Staining and Bone Resorption Assay

RAW 264.7 cells were purchased from the Cell Bank of the Chinese Academy of Medical Sciences. Bone marrow monocytes (BMMs) isolated from Cko and Ctrl mouse femurs and tibias were treated with 100 ng/ml receptor activator of nuclear factor-κB ligand (RANKL; R&D Systems) and 30 ng/ml mouse macrophage colony-stimulating factor (M-CSF; R&D Systems) for 6 days. All the serum- or supplement-containing media were replaced every 2 days. Cells were exposed to hypoxia by incubation under 2% O_2_ and 5% CO_2_ conditions with balanced N_2_ in the Invivo2 Hypoxia Workstation (Rushkinn, Waltham, MA). BMMs were treated under hypoxic conditions for the last 48 h. The cells were stained with a TRAP staining kit, toluidine blue staining kit (Solarbio) and FITC phalloidin (Sigma-Aldrich). Osteoclasts were identified by TRAP staining, and TRAP-positive osteoclasts were counted in multiple sections. For the bone resorption assay, BMMs were seeded on bone slices in 24-well plates and cultured with osteoclastogenic medium for 14 days. The medium was aspirated, and sodium hypochlorite was used to bleach the samples three times. Those methods are the same as our previous study (Tian et al., 2020).

### 2.3 X-Ray and Micro-computed Tomography

X-ray and computed tomography were used to quantify bone remodelling by using a micro-CT system (Micro-CT 50, Scanco Medical, United States) and the related analysis software. We selected the entire bone defect region for analysis, and 40 Z planes were imaged. Five male mice from the Ctrl or Cko group were euthanized by 1% pentobarbital sodium i.p. at 8 weeks, and the mandibles were collected for micro-CT analysis. Statistical analysis of condylar head width and bone volume fraction was conducted.

### 2.4 Staining

Sections were stained with an H&E Staining Kit (KeyGen) and TRAP staining kit (Sigma-Aldrich). An alkaline phosphatase (ALP) staining kit (Jiancheng) was utilized to detect bone formation. For immunofluorescence staining, the primary antibodies used were rabbit anti-HIF-1α (1:200; Abcam) and mouse anti-CTSK (1:200; BBI). Then, the slides were incubated with DyLight 488-conjugated goat anti-mouse IgG (1:500; Abbkine) or DyLight 594-conjugated goat anti-rabbit IgG (1:500; Abbkine) followed by DAPI (1:800; Sigma-Aldrich).

### 2.5 RNA Isolation and qRT-PCR

BMMs were seeded in 6-well plates as previously described and replicated three times. Then, RNA was isolated from cultured cells or mandibular tissue with TRIzol Reagent (TaKaRa, Japan) according to the manufacturer’s instructions. The purity and concentration of the RNA samples were determined with a NanoDrop 2000 spectrometer (NanoDrop Technology, United States). First-strand cDNA was transcribed from 1,000 ng RNA with the PrimeScript RT reagent Kit with gDNA Eraser (TaKaRa, Japan). qRT-PCR was performed in triplicate with Applied Biosystems QuantStudio 6 and a SYBR Premix Ex Taq II kit (TaKaRa). The primer sequences are shown in Supplementary Table S1. The amplification conditions were as follows: 30 s at 95°C, followed by 45 cycles of 10 s at 95°C and 35 s at 62°C. The primers used for qRT-PCR were purchased from Sango Biotech (Shanghai, China). β-Actin expression was used for normalization, and mRNA expression was calculated using the 2-ΔΔCT method.

### 2.6 RNA-Seq Analysis

Total RNA was extracted from Cko and Ctrl mouse bone marrow macrophages after the cells were treated with RANKL (100 ng/ml) and M-CSF (30 ng/ml) for 6 days. The RNA-seq analysis was performed by Personalbio Co. (Shanghai, China) used by Illumina HiSeq. The first-strand cDNA was synthesized using RNA as a template, using 6-base random primers and reverse transcriptase, and the second-strand cDNA was synthesized using the first-strand cDNA as a template. After the library was constructed, PCR amplification was used to enrich the library fragments, and then the library was selected according to the fragment size, and the library size was 450bp. After RNA extraction, purification, and library construction of the samples, the next-generation sequencing technology was used to perform paired-end (PE) sequencing on these libraries based on the Illumina HiSeq sequencing platform. To obtain lncRNAs, we screened transcripts with a length greater than or equal to 200 bp and the number of exons greater than or equal to 2. Next, we screen for transcripts whose Class_code is x/u/i (x refers to the anti-strand of the reference transcript, u represents the unknown transcript, and i is completely in an intron of the reference transcript). Finally, we screened the lncRNAs that appeared in the same sample more than 3 times, and finally obtained lncRNAs. Based on the expression profiles of 6 groups of samples (including three control groups and three CKO groups), we used the DEseq R package to perform differential analysis of gene expression, and the screening criteria were set as |log2(Fold change) |>1 and *p*-value< 0.05). After predicting the target genes of lncRNA, we used Cytoscape to construct a co-expression network of lncRNA-mRNA and select the key lncRNAs. According to the target genes of key lncRNAs, we constructed a protein interaction network through the STRING database and visualized it with Cytoscape. Topological analysis can identify the core proteins in the protein interaction network and provide directions for future research.

### 2.7 Protein Extraction and Western Blotting Assay

RAW 264.7 cells were seeded in 60-mm dishes and incubated with PBS or in a hypoxic environment for 24 h. The cells were washed three times with PBS and then harvested in RIPA buffer with protease inhibitors on ice. The supernatants were collected, stored at −80°C, and then centrifuged at 12,000 rpm for 10 min at 4°C. The protein concentrations were determined by a BCA protein assay kit (Thermo, United States). The protein samples were separated by 6%–10% sodium dodecyl sulfate-polyacrylamide gel electrophoresis (SDS-PAGE) and then transferred to polyvinylidene fluoride (PVDF) membranes (Sigma-Aldrich, United States). The PVDF membranes cut prior to hybridisation with antibodies. The PVDF membranes were incubated for 1 hour with 5% non-fat milk to block nonspecific binding. Then, the PVDF membranes were incubated with primary antibodies against HIF-1α and β-actin at 4°C overnight. After washing 3 times with TBST for 10 min, the membranes were incubated with HRP-conjugated anti-rabbit or anti-mouse antibodies at 37°C for 1 h followed by washing with TBST 3 times for 10 min. Then, the membranes were visualized with the Super-signal West-Pico chemiluminescent substrate (Thermo Scientific, United States). The band intensities were analysed by a Smart ChemTM Image Analysis System (Sagecreation, China). The protein bands were scanned and quantified by ImageJ software. Those methods are the same as our previous study (Tian et al., 2020).

### 2.8 GO and KEGG Pathway Analyses

GO analysis was performed using KOBAS 3.0 software (available online: http://kobas.cbi.pku.edu.cn), and the analysis included three domains: cellular component (CC), molecular function (MF) and biological process (BP). GO analysis provides label classification of gene function and gene product attributes (http://www.geneontology.org). KEGG pathway analysis identified the significant enrichment of different pathways with KOBAS 3.0 software (http://www.genome.jp/kegg) (Kanehisa et al., 2021). We used red lines show the important part concerning of osteoclasts.

### 2.9 Co-expression Network Construction

Based on the expression profiles of 6 groups of samples (including three control groups and three CKO groups). The co-expression network was established by calculating the Pearson correlation coefficient and *p*-value between multiple genes. In this study, the transcripts were filtered using a COR of >0.85 and a *p*-value of <0.05. The co-expression network was illustrated using Cytoscape software.

### 2.10 Statistical Analysis

Statistical analysis was conducted with SPSS 22.0 software (Chicago, IL, United States). Student’s t test was used for comparisons between two groups. A *p*-value of <0.05 was considered significant.

## 3 Results

### 3.1 HIF-1α Increased Osteoclastogenesis and Bone Resorption

**FIGURE 1:**
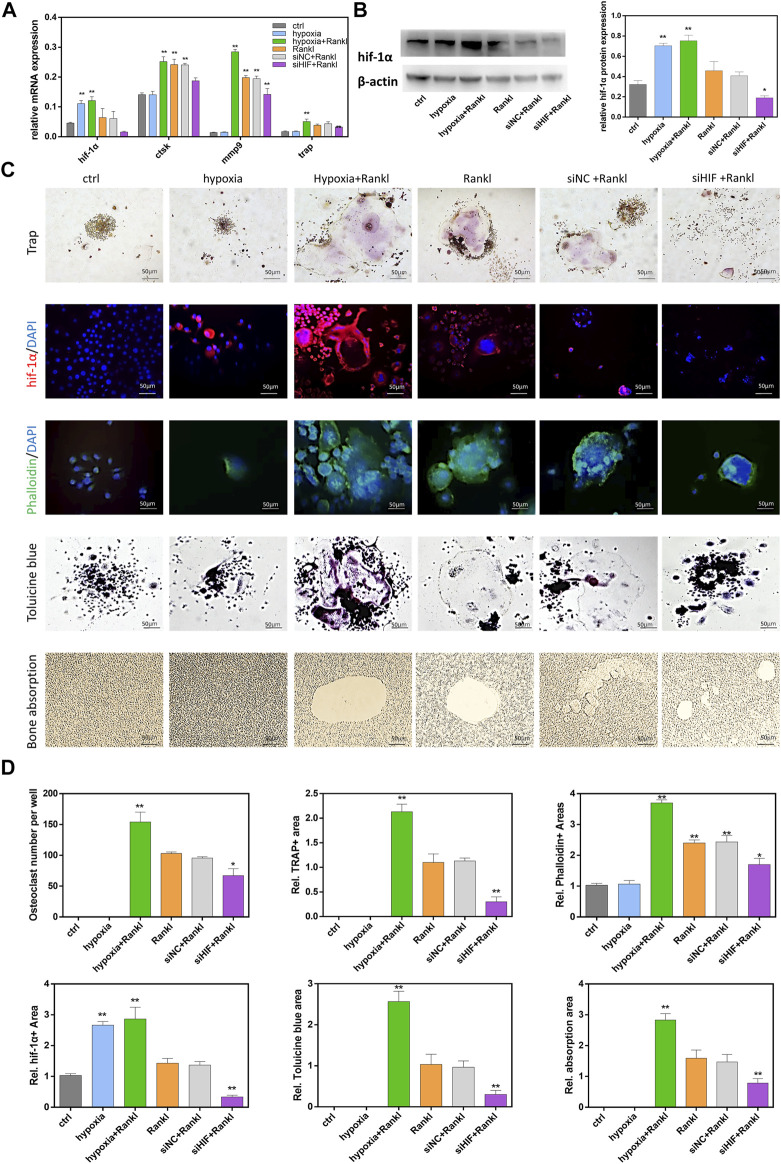
HIF-1α increased osteoclastogenesis and bone resorption. **(A)**The expression of ctsk, trap, mmp9, hif-1α mRNA in RAW264.7 cells. RAW264.7 cells were treated with hypoxia in the presence or absence of siRNA transfection. **(B)**The expression of hif-1α protein in RAW264.7 cells. RAW264.7 cells were treated with hypoxia in the presence or absence of siRNA transfection. Representative images of western blot: Hif-1α protein expression and analysis. The blots cut prior to hybridisation with antibodies. **(C)**The staining of Trap, HIF-1α, Phalloidin, Tolucine blue in RAW264.7 cells that were treated hypoxia in the presence or absence of siRNA transfection. And the images of bone absorption. **(D)** Statistical analysis of number of osteoclasts the area of Trap, HIF-1α, Phalloidin, Tolucine blue and bone absorption. Bars = 50 μm*, *p* < 0.05, **, *p* < 0.01. Data represent mean ± SEM.

Induced by RANKL, RAW264.7 cells could differentiate into multinucleated TRAP-positive osteoclasts and they were capable of resorbing bone. To determine the function of HIF-1α in osteoclasts during osteoclastogenesis and bone resorption, a hypoxic environment was used to increase HIF-1α expression, and siRNA was used to decrease HIF-1α expression. Osteoclast-related gene (ctsk, trap, mmp9) transcription was quantified by qRT-PCR, and HIF-1α protein expression was quantified by western blotting (Figures 1A,B). Increased expression of HIF-1α was accompanied by increased expression of osteoclast-related genes. In addition, osteoclast-related gene expression was decreased when the expression of HIF-1α was inhibited by siHIF. However, HIF-1α mRNA and protein expression did not change after cells were incubated with or without RANKL. In addition, phalloidin staining and toluidine blue staining showed that under hypoxic conditions, the number of osteoclasts increased, the efficacy of siHIF decreased, and the area of HIF-1α expression increased. These results indicated that HIF-1α was involved in osteoclast activation (Figures 1C,D). To explore the role of HIF-1α in bone resorption, RAW 264.7 cells were seeded onto bone slices. The results showed no bone absorption without RANKL treatment. Compared with the control, after osteoclastogenesis, the absorbed area increased under hypoxic conditions and decreased with siHIF treatment (Figures 1C,D). These results confirmed that HIF-1α played a key role in osteoclast bone resorption *in vitro*.

### 3.2 CKO Mouse Identification and Osteoclast Phenotype Assessment

**FIGURE 2:**
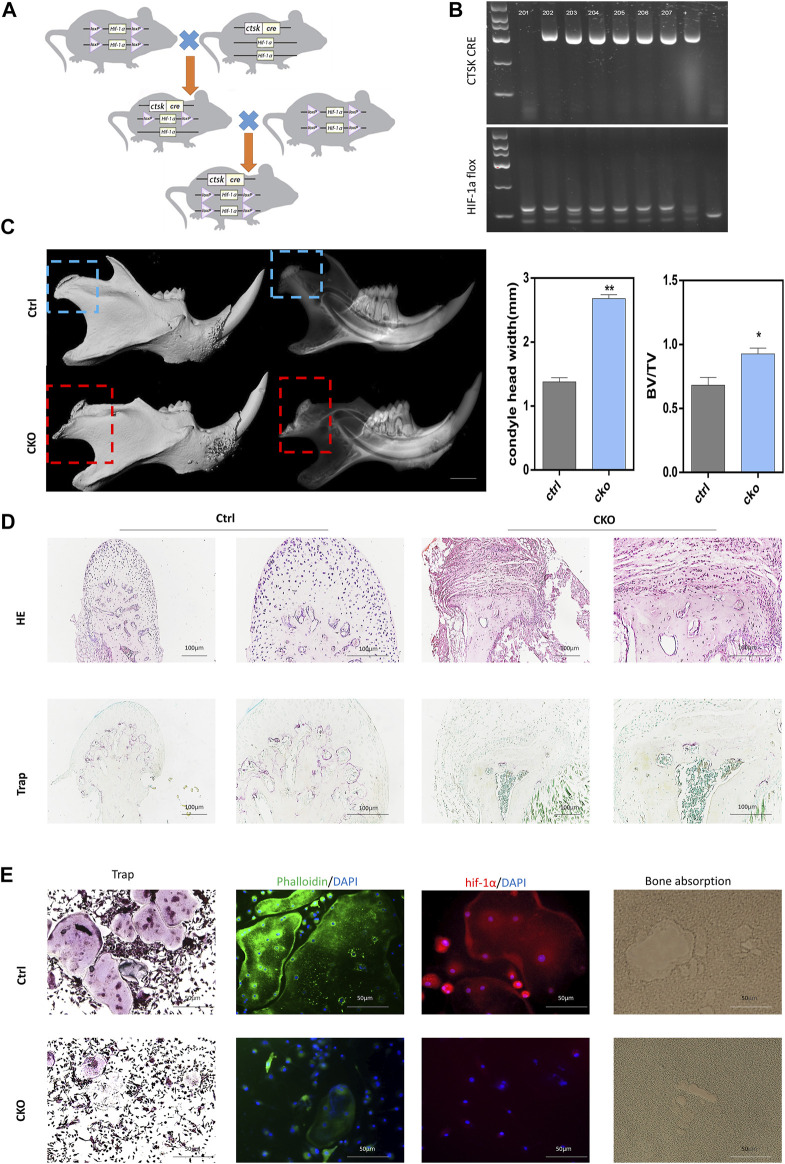
The phenotype of knock out HIF-1α in osteoclast. **(A)**Model mouse breeding pattern diagram of conditional knockout HIF-1α in osteoclast. **(B)** The gene identification of Ctrl and Cko mouse. **(C)**Typical phenotype after knock out HIF-1α in osteoclast:the 3D construction and X-ray of condyle dysplasia. Statistical analysis of condyle head width and Bone volume fraction (BV/TV), (n = 5). Bars = 1.0 mm*, *p* < 0.05, **, *p* < 0.01. Data represent mean ± SEM. **(D)**. The representative staining images of HE, Trap of condyle dysplasia. Bars = 100 μm. **(E)** The representative staining images of HE,Trap, HIF-1α, Phalloidin, bone absorption in Ctrl osteoclasts and Cko osteoclasts. Bars = 100 μm.

After HIF-1α flox and Ctsk cre gene identification (Figures 2A,B), we allocated HIF-1αflox/flox; Ctsk cre + mice to the CKO group and the other mice to the Ctrl group. First, we compared the hard tissues using micro-CT. Interestingly, we found that the condylar dysplasia (Figure 2C) and condylar head width of the CKO mice were twice as wide as those of the Ctrl mice, and the bone volume fraction (BV/TV) in the CKO mice was also higher. These results indicated that HIF-1α played an essential role in osteoclast bone resorption *in vivo*. With HE staining, we found that the shape of the condyles of the CKO mice changed from oval to flat, and TRAP staining showed that the number of osteoclasts decreased significantly (Figure 2D). Then, to determine the phenotype of HIF-1α-knockout osteoclasts and the function of HIF-1α in osteoclasts during osteoclastogenesis and bone resorption, we used M-CSF and RANKL to induce BMMs extracted from Cko and Ctrl mouse bone marrow to differentiate into osteoclasts. There were more multinucleated and TRAP-positive cells in the Ctrl group than in the Cko group (Figure 2E). As expected, the areas of phalloidin staining, HIF-1α expression and bone resorption were smaller in the Cko group. These results confirmed that HIF-1α was vital for osteoclast bone resorption *in vitro*.

### 3.3 mRNA Expression Profiles in HIF-1α-Knockout Osteoclasts and Pathway Analysis

**FIGURE 3:**
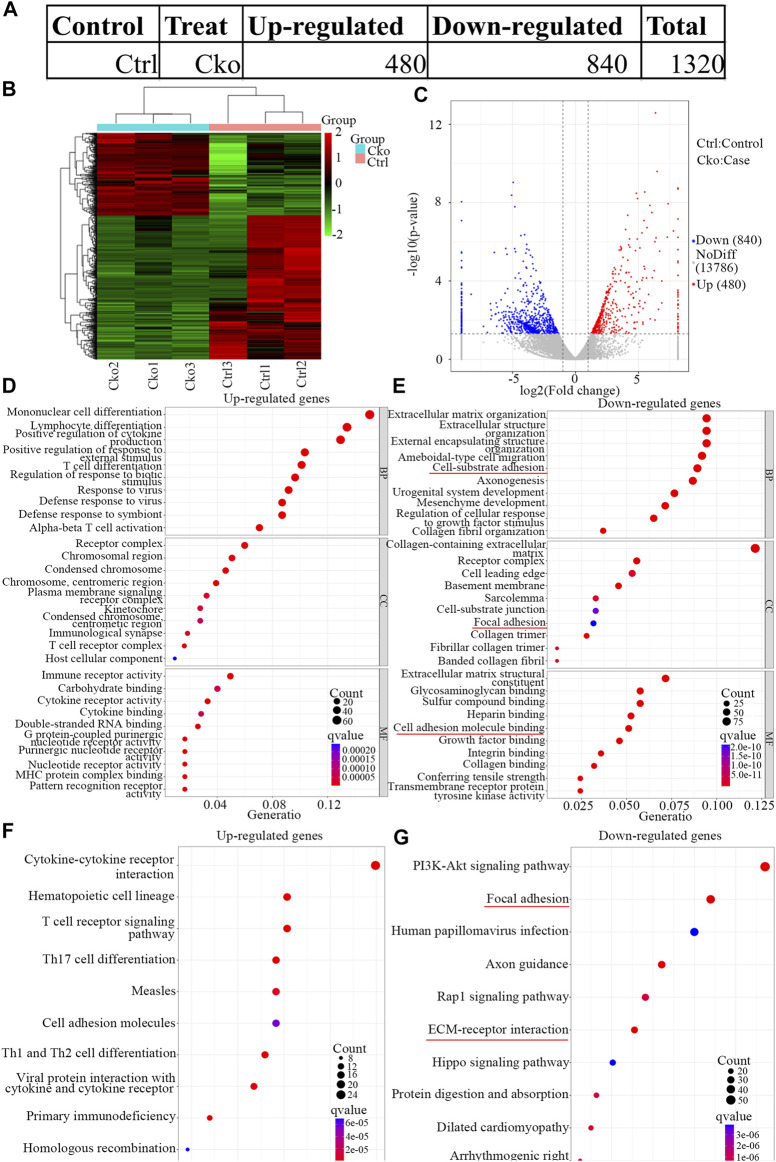
Expression profiles of mRNAs in knock out HIF-1a osteoclast and pathway analysis. The up-regulated and down-regulated number of mRNA **(A)** Cluster heat map shows differentially expressed mRNAs **(B)** in osteoclast differentiation. The names of the sample groups are on the *x*-axis and the different profiles are on the *y*-axis. The red strip indicates high relative expression and the green strip indicates low relative expression. Volcano plots of mRNAs **(C)** in Ctrl versus Cko osteoclasts. Red squares in the plots represent the up-regulated transcripts with statistical significance while blue squares represent the down-regulated transcripts (Fold change ≥2.0, *p* < 0 0.05). **(D,E)** Top 30 GO terms with significantly differential up and down-expression from GO analysis of molecular function, biological process and cellular component are shown in histogram. **(F,G)** Top 10 KEGG pathways (Kanehisa et al., 2021) with significantly differential up and down-expression Red lines show the important part concerning of osteoclasts.

To explore the changes in mRNA expression after HIF-1α expression was knocked out in osteoclasts, mRNA expression in Cko or Ctrl osteoclasts was analysed by RNA-seq. A total of 1,320 mRNAs were changed (Figure 3A), and 480 mRNAs were more highly expressed in the Ckogroup. Moreover, 840 mRNAs were expressed at lower levels in the Cko group, as shown in the heatmap (Figure 3B) and the volcano plot (Figure 3C). The top ten up- or downregulated mRNAs are shown in Supplementary Table S2. The top 30 significantly enriched GO terms are presented in Figures 3D,E. GO analysis showed that typical up-regulated genes GO terms were Mononuclear cell differentiation, Receptor complex and Immune receptor activity. Typical down-regulated genes GO terms were Extracellular matrix organization, Collagen-containing extracellular and Extracellular matrix structural constituent. GO terms of multicellular organismal process, system development and developmental process changed distinctively, and HIF- 1α might be key for bone metabolism in osteoclasts. KEGG analysis showed that 279 KEGG terms were changed in HIF-1α-knockout osteoclasts. The top 10 significantly enriched KEGG terms are presented in Figures 3F,G. Of the pathways affected by changes in mRNA expression after HIF-1α expression was knocked out, the focal adhesion and ECM-receptor interaction pathways might be the most important. Vinculin (Fukunaga et al., 2014) which plays a key role in focal adhesion pathways, had been confirmed drive the osteoclast phenotype.

### 3.4 LncRNAs Expression Profiles in HIF-1a-Knockout Osteoclasts and Pathway Analysis

**FIGURE 4:**
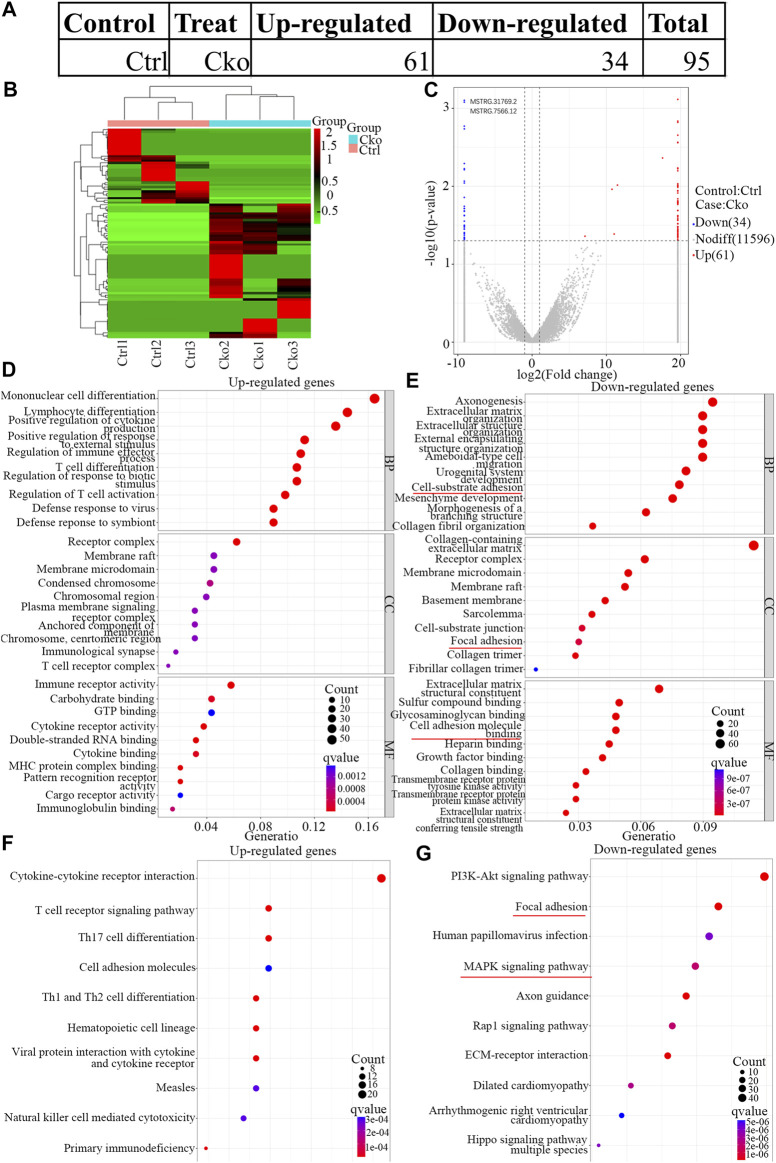
Expression profiles of LncRNAs in knock out HIF-1a osteoclast and pathway analysis. The up-regulated and down-regulated number of LncRNA **(A)** Cluster heat map shows differentially expressed LncRNAs **(B)** in osteoclast differentiation. The names of the sample groups are on the *x*-axis and the different profiles are on the *y*-axis. The red strip indicates high relative expression and the green strip indicates low relative expression. Volcano plots of LncRNAs **(C)** in Ctrl versus Cko osteoclasts. Red squares in the plots represent the up-regulated transcripts with statistical significance while blue squares represent the down-regulated transcripts (Fold change ≥2.0, *p* < 0 0.05). **(D,E)** Top 30 GO terms with significantly differential up and down-expression from GO analysis of molecular function, biological process and cellular component are shown in histogram. **(F,G)** Top 10 KEGG pathways (Kanehisa et al., 2021) with significantly differential up and down-expression Red lines show the important part concerning of osteoclasts.

To identify the changes in lncRNA expression after HIF-1α expression was knocked out in osteoclasts, lncRNAs expression in Cko or Ctrl osteoclasts was analysed by RNA-seq. A total of 95 lncRNAs were differentially expressed (Figure 4A), and 61 lncRNAs were expressed at higher levels in the Cko group. Moreover, 34 lncRNAs were downregulated in the Cko group, as shown in the heatmap (Figure 4B) and the volcano plot (Figure 4C). The top ten up- or downregulated lncRNAs are shown in Supplementary Table S3. GO analysis showed that typical up-regulated genes GO terms were Mononuclear cell differentiation, Receptor complex and Immune receptor activity. Typical down-regulated genes GO terms were Axonogenesis, Collagen-containing extracellular matrix and Extracellular matrix structural constituent. The top 30 significantly enriched GO terms are presented in Figure 4D E. KEGG analysis showed that a total of 99 KEGG terms were differentially expressed in HIF-1α-knockout osteoclasts, and many genes enriched in the MAPK pathway changed after knockout of HIF- 1α in osteoclasts. The top 10 significantly enriched KEGG terms are presented in Figures 4F,G. Of the pathways affected by changes in lncRNA expression after HIF-1α expression was knocked out, the PI3K-Akt signaling pathway and MAPK signaling. The MAPK pathway (Lin et al., 2021) has been confirmed to be inseparable for osteoclast development and maturation. Thus, MAPK pathway might be the most important after knockout of HIF-1α, and these pathways might drive the osteoclast phenotype.

### 3.5 Construction of a lncRNA-mRNA Co-expression Network

**FIGURE 5:**
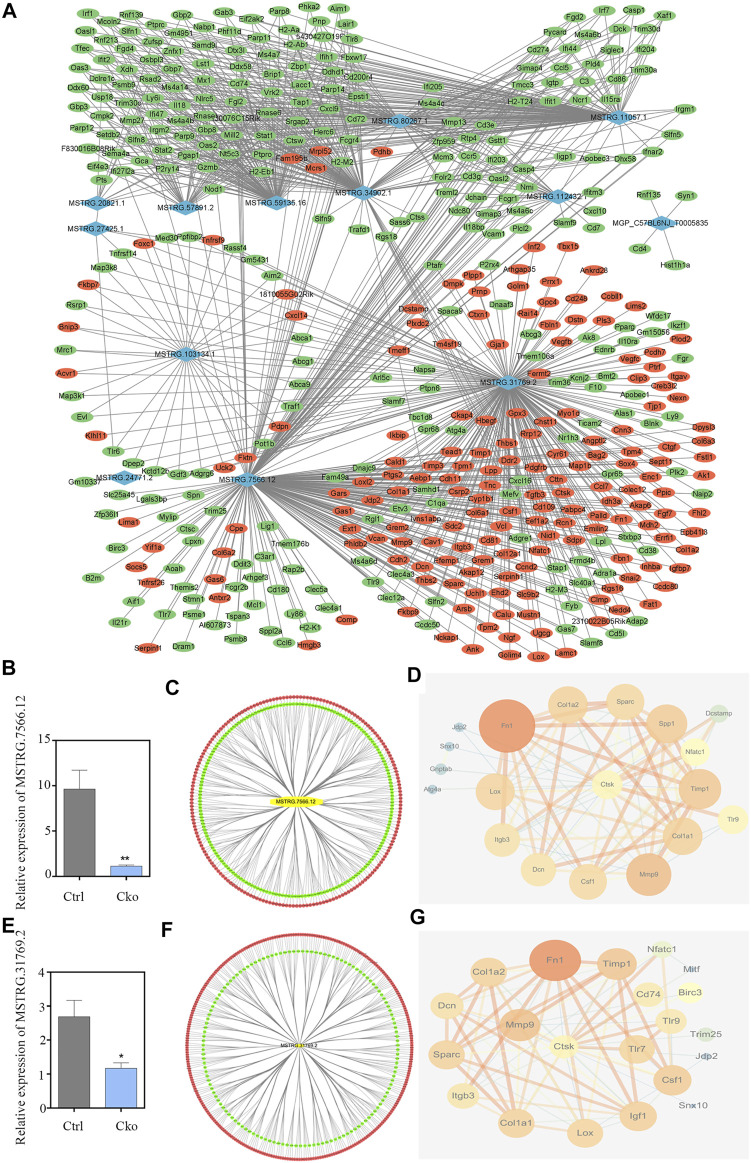
Construction of the lncRNA-mRNA co-expression and PPI network. **(A)**Construction of the lncRNA-mRNA co-expression network. Up-regulated lncRNAs are shown in purple and down-regulated lncRNAs are shown in blue. Up-regulated mRNAs are shown in red and down-regulated mRNAs are shown in green. **(B)** The expression of lncRNA MSTRG.7566.12 in Ctrl and Cko osteoclasts. **(C)** Prediction of the lncRNA MSTRG.7566.12 mRNA co-expression network. Up-regulated mRNAs are shown in red and down-regulated mRNAs are shown in green. **(D)** Prediction of the lncRNA MSTRG.7566.12 and osteoclasts protein protein interaction network. **(E)** The expression of lncRNA MSTRG.31769.2 in Ctrl and Cko osteoclasts. **(F)** Prediction of the lncRNA MSTRG.31769.2 and mRNA co-expression network. Up-regulated mRNAs are shown in red and down-regulated mRNAs are shown in green. **(G)** Prediction of the lncRNA MSTRG.31769.2 and osteoclasts protein protein interaction network.

**FIGURE 6:**
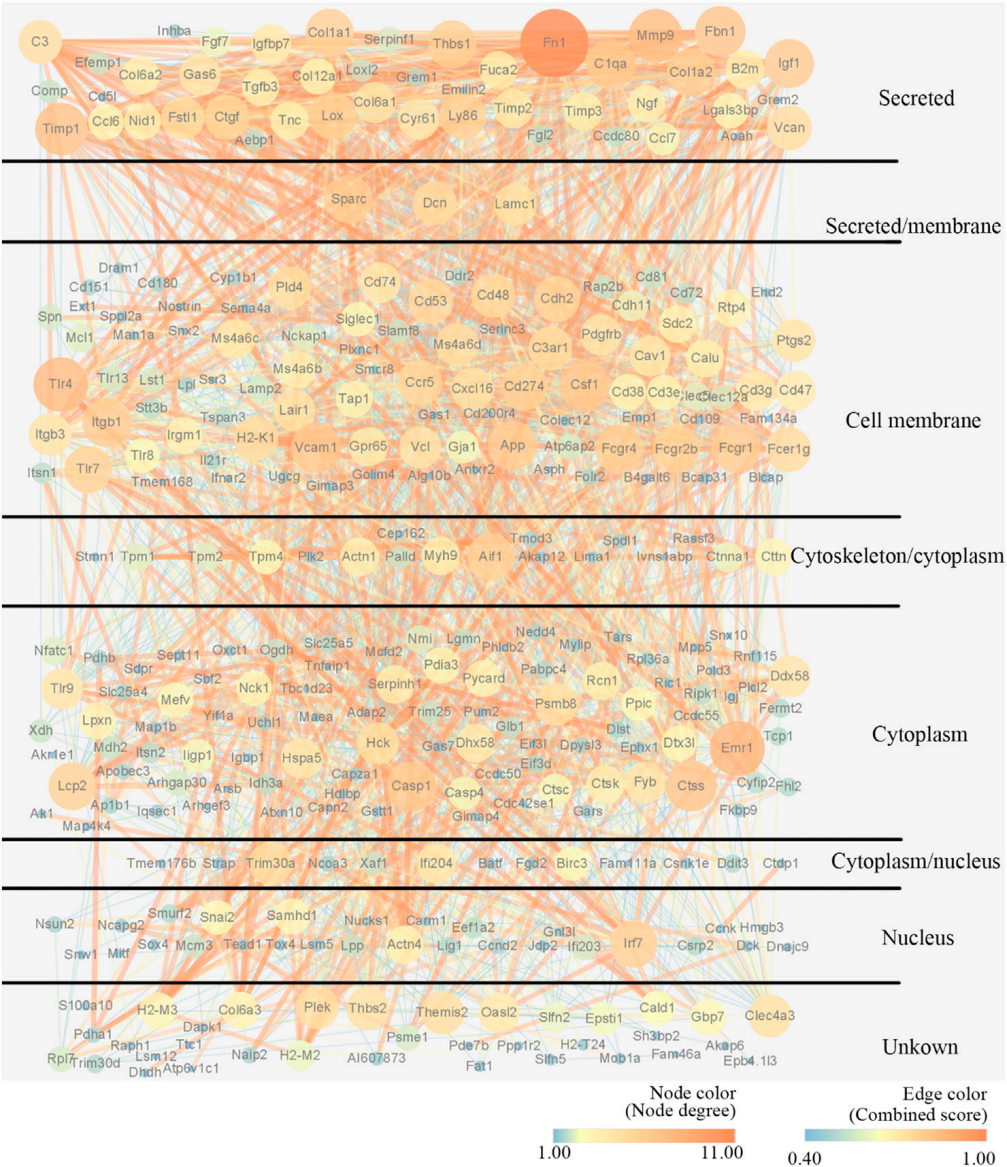
Construction of the PPI network and potential location of osteoclast. Nodes represent proteins. The strings connect the co-expression proteins. The size of each circle is determined by the number of other proteins that interact with these proteins.

To explore the potential interactions between mRNAs and lncRNAs, a lncRNA-mRNA co-expression network was established. By searching for protein-coding genes within 10 KB upstream and downstream of LncRNA genes, thirteen lncRNAs were selected from the differentially expressed lncRNAs and further built as 416 pairs of co-expressed lncRNAs-mRNAs (Figure 5A). In the co-expression network, the two lncRNAs with the two highest numbers of interactions were lncRNA MSTRG.31769.2 and MSTRG.7566.12. The expression of lncRNA MSTRG.7566.12 was confirmed by qRT-PCR, and its correlations with the expression levels of other molecules were analysed by Pearson correlation coefficient (Figures 5B,C). The protein-protein interaction network results showed that lncRNA MSTRG.7566.12 expression had a strong negative correlation with Mmp9 and Ctsk expression (Figure 5D). The expression of lncRNA MSTRG.31769.2 was confirmed by qRT-PCR, and its correlations with the expression levels of other molecules were analysed by Pearson correlation coefficient (Figures 5E,F). The protein-protein interaction network results showed that MSTRG.31769.2 expression had a strong negative correlation with Mmp9 and Ctsk expression (Figure 5G). The protein interaction network and potential location in osteoclasts are shown in Figure 6. In the secreted part, Fn1 and Mmp9 were dramatically enriched, which indicated that after knocking out HIF-1α, these proteins would eventually be affected by many upstream proteins. These results indicated that HIF-1α knockout changed the lncRNA-mRNA interaction networks and regulated osteoclast metabolism.

## 4 Discussion

In our study, by changing the expression of HIF-1α in RAW 264.7 cells, we found that HIF-1α could promote the differentiation of these cells into osteoclasts. When HIF-1α was expressed at low levels in RAW 264.7 cells, there were fewer multinucleated TRAP + osteoclasts. Moreover, the bone resorption function of RAW 264.7 cells was also weak. All these results indicated that HIF-1α was involved in the differentiation of osteoclasts and the bone resorption function of osteoclasts *in vitro* (Sun et al., 2015). Then, we attempted to determine whether HIF-1α was related to the differentiation of osteoclasts and the bone resorption function of osteoclasts. *In vivo*, we bred HIF-1αflox/flox; Ctsk cre + mice to specifically knock out HIF-1α in osteoclasts. We found that the condyle was abnormal, and this phenotype was thoroughly studied in our previous work (Tian et al., 2020; Tang et al., 2020). In this study, we isolated and cultured monocytes from CKO and Ctrl mice to induce osteoclast differentiation. We found that there was fewer multinucleated TRAP + osteoclasts in the CKO cultures than in the Ctrl cultures. Moreover, the bone resorption area of osteoclasts in the CKO group was smaller than that in the Ctrl group. Through RNA-seq analysis, a large number of differentially expressed lncRNAs and mRNAs were identified in HIF-1α-knockout osteoclasts. Then, the cellular events and biological pathways in HIF-1α-knockout osteoclasts were identified with GO and KEGG analysis. Christian et al. verified that osteoclasts ensured remodelling of the bone and its haematopoietic niche (Jacome-Galarza et al., 2019). We also found that GO terms of multicellular organismal process, system development and developmental process changed distinctively, and HIF- 1α might be key for bone metabolism in osteoclasts. The MAPK pathway has been confirmed to be inseparable for osteoclast development and maturation (Koga et al., 2019; Kumar et al., 2021). Our experiments showed that many genes enriched in the MAPK pathway changed after knockout of HIF- 1α in osteoclasts. In addition, the co-expression network revealed interactions between mRNAs and lncRNAs, as well as the core regulator, in HIF-1α-knockout osteoclasts. Eventually, two lncRNAs (MSTRG.31769.2 and MSTRG.7566.12) were identified that might play a central role in HIF-1α-knockout osteoclasts, and these lncRNAs might influence HIF-1α expression and regulate osteoclast metabolism. Li et al. (2017) reported that ROR1-HER3-lncRNA could regulate bone metastasis. Our study provides a basis for further understanding the role and mechanism of lncRNAs (MSTRG.31769.2 and MSTRG.7566.12) in HIF-1α-knockout osteoclasts, and many of these molecules are potential targets for treating bone metabolism diseases, such as condylar dysplasia. As the Ras signalling pathway was distinctly enriched, the HIF-1α- Ras -lncRNA axis might regulate bone metabolism. Prediction of the lncRNA and protein-protein interaction network indicated that lncRNAs might influence the csf1, ctsk and mmp9 proteins which are indispensable for osteoclast function. We speculated that lncRNA MSTRG. 7566.12 and lncRNA MSTRG.7566.12 had potential significance for osteoclast function. After knockout HIF-1α, some key functional proteins of osteoclasts, such as csf1, ctsk and mmp9 will be affected.

Osteoclasts are multinucleated cells that absorb calcified matrix by secreting acid and collagen-degrading enzymes (Roodman, 1996). HIF-1α plays a crucial role in key stages of metastatic dissemination, including angiogenesis, epithelial-mesenchymal transition, invasion, cancer stem cell maintenance, tumour cell dormancy, extracellular vesicle release, and pre-metastatic niche generation (Semenza et al., 1991; Lee et al., 2004; McGettrick and O’Neill, 2020). HIF-1α also affects bone cells, such as osteoblasts, chondrocytes and osteoclasts, and immune cells, which also perform vital functions in supporting bone metastasis (Devignes et al., 2018; Stegen et al., 2018; Stegen et al., 2019). Over the decades, numerous studies have confirmed that HIF-1α plays a crucial role in osteoclast development and bone resorption by regulating nutrient and energy sensors. For example, osteoclast-mediated bone destruction is enhanced in the hypoxic synovial microenvironment in rheumatoid arthritis (Knowles, 2019). The increase in bone resorption in rheumatoid arthritis is driven by the hypoxia-inducible transcription factor HIF-1α. Inactivation of HIF-1α can antagonize bone loss in ovariectomized (Ovx) mice and osteoclast-specific oestrogen receptor α-deficient mice (Miyauchi et al., 2013). An oral HIF-1α inhibitor exerts a protective effect on osteoclast activation and bone loss in Ovx mice. In addition, HIF-1α promotes the expression of RANKL in MLO-Y4 cells by activating the JAK2/STAT3 pathway and enhances the osteoclast-mediated differentiation of osteoclasts (Zhu et al., 2019). Hypoxia-induced glycolysis in osteoclasts is an adaptive mechanism of alveolar bone remodelling after mandibular osteotomy.

Moreover, an increasing number of studies have demonstrated that osteoclast differentiation is precisely regulated by lncRNAs, such as lncRNA ENSG00000257764.2 (Liu et al., 2020), lncRNA Nron (Zhang et al., 2020a), and lncRNA Neat1 (Zhang et al., 2020b). Recently, many studies have focused on a regulatory feedback loop between HIF-1α and several lncRNAs. For instance, a HIF-1α-specific lncRNA can directly regulate the stability of HIF-1α mRNA and thus confer poor prognosis in lung cancer (Hua et al., 2020). LncRNAs indirectly regulate HIF-1α expression by interfering with the proteasome degradation mechanism that regulates HIF-1α expression (Barth et al., 2020). HIF-1α stable long non-coding RNA (HISLA) enhances aerobic glycolysis and apoptosis resistance in breast cancer cells (Chen et al., 2019). HISLA inhibits the hydroxylation and degradation of HIF-1α by blocking the interaction between PHD2 and HIF-1α. In turn, the lactic acid released by glycolytic tumour cells upregulates HISLA expression in macrophages, forming a feed-forward loop. Our study carefully examined HIF-1α, lncRNAs and osteoclasts. We tried to determine the relationship between HIF-1α and lncRNAs in osteoclasts, especially whether lncRNAs play a role in the regulation of HIF-1α protein levels. This study provides a new opportunity for an in-depth understanding of the mechanism by which lncRNAs interact with HIF-1α, which might play a key role in condyle development and bone metabolism.

In summary, we explored lncRNA-mRNA expression patterns in the differentiation of osteoclasts by RNA sequencing and identified possible regulatory mechanisms by analysing biological data. The aim was to clarify the role of lncRNA in both bone differentiation and osteoblast anomalies. Our results may help create a potential target for abnormal bone metabolism and shed light on the mechanism of osteoclasts differentiation. However, our study still has some limitations, including a lack of studies on lncRNA function to indicate these differences. Further research is needed to examine the role of these differentially expressed lncRNAs in osteoclast differentiation.

## Data Availability

The datasets presented in this study can be found in online repositories. The names of the repository/repositories and accession number(s) can be found in the article/Supplementary Material.

## Supplementary Material

Supplementary Table S1 Genes and corresponding primers.

Supplementary Table S2 Top ten upregulated and downregulated mRNAs.

Supplementary Table S3 Top ten upregulated and downregulated LncRNAs.

Supplementary Table S4 GO analysis.

Supplementary Table S5 GO term analysis.

Supplementary Table S6 KEGG analysis.

Supplementary Table S7 PCR analysis.

Supplementary Table S8 high resolution figure.

Supplementary Table S9 gene notation.

Supplementary Table S10 gene expression.
