# Supplementary material for: LncRNA-mRNA Expression Profiles of Osteoclast After Conditional Knockout HIF-1α
Source: Front Genet. 2022 Jun 21;13:909095. doi: 10.3389/fgene.2022.909095 (PMC9253292; doi:10.3389/fgene.2022.909095)
Supplement: Supplementary file 3 [file Table8.DOCX]

**Supplement table 1 Genes and corresponding primers**

| **Gene name** | **Forward/Reverse** | **Sequence (from 5' to 3')** |
| --- | --- | --- |
| ctsk | forward | CTTCCAATACGTGCAGCAGA |
| ctsk | reverse | TCTTCAGGGCTTTCTCGTTC |
| trap | forward | CACTCCCACCCTGAGATTTGT |
| trap | reverse | CCCCAGAGACATGATGAAGTCA |
| mmp9 | forward | CAAAGACCTGAAAACCTCCAA |
| mmp9 | reverse | GGTACAAGTATGCCTCTGCCA |
| HIF-1α | forward | GAATGAAGTGCACCCTAACAAG |
| HIF-1α | reverse | GAGGAATGGGTTCACAAATCAG |
| β-actin | forward | GAAATCGTGCGTGACATCAAA |
| β-actin | reverse | TGTAGTTTCATGGATGCCACAG |
| MSTRG.7566.12 | forward | CCCTCACTGCTCTCTCTGTCCT |
| MSTRG.7566.12 | reverse | CATGACCATTGCTCATCCCTC |
| MSTRG.31769.2 | forward | CAGGTGTTGACGCGATGTGA |
| MSTRG.31769.2 | reverse | TAAGTGGGAAGCCCCAGTG |

**Supplement table 2 Top ten upregulated and downregulated mRNAs**

| TOP10 Up mRNA | TOP10 Down mRNA | |
| --- | --- | --- |
| Gimap4 | Mt3 |  |
| Ikzf3 | Cyp2s1 |  |
| Themis | Capn6 |  |
| Cd7 | Scin |  |
| Gimap3 | Slc6a17 |  |
| Jchain | Edil3 |  |
| Gm4951 | Rufy4 |  |
| Ncr1 | Sema7a |  |
| Cd3g | Prkcdbp |  |
| Cd3e | Kank1 |  |

**Supplement table 3 Top ten upregulated and downregulated LncRNAs**

| TOP10 Up LncRNA | TOP10 Down LncRNA |
| --- | --- |
| MSTRG.1990.9 | MSTRG.1990.8 |
| MSTRG.1990.13 | MSTRG.41781.2 |
| MSTRG.1990.14 | MSTRG.77697.2 |
| MSTRG.1990.6 | MSTRG.1990.17 |
| MSTRG.46899.2 | MSTRG.1990.21 |
| MSTRG.1990.11 | MSTRG.23904.1 |
| MSTRG.1990.7 | MSTRG.1990.5 |
| MSTRG.1990.4 | MGP_C57BL6NJ_T0001556 |
| MSTRG.1990.15 | MSTRG.1990.16 |
| MSTRG.33517.1 | MSTRG.24308.3 |

**Supplement figure 1 Original Figure of Figure 1B**

**
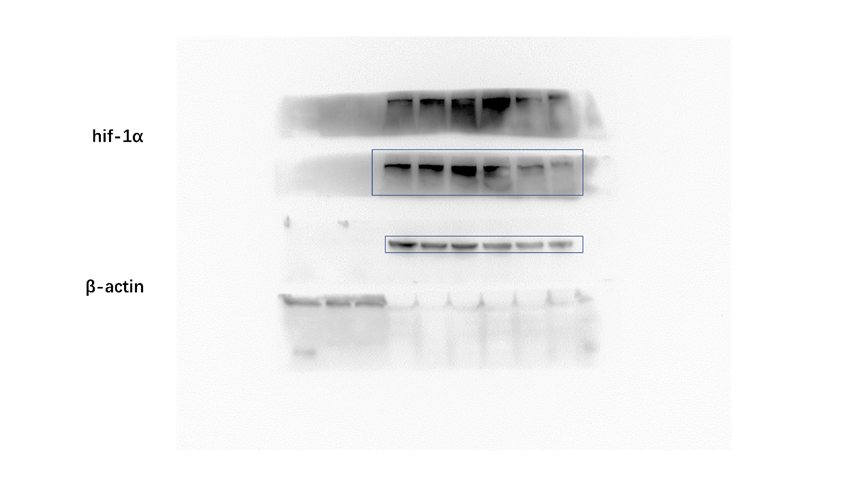
**

**Supplement figure 2 Original Figure of Figure 2B**

**
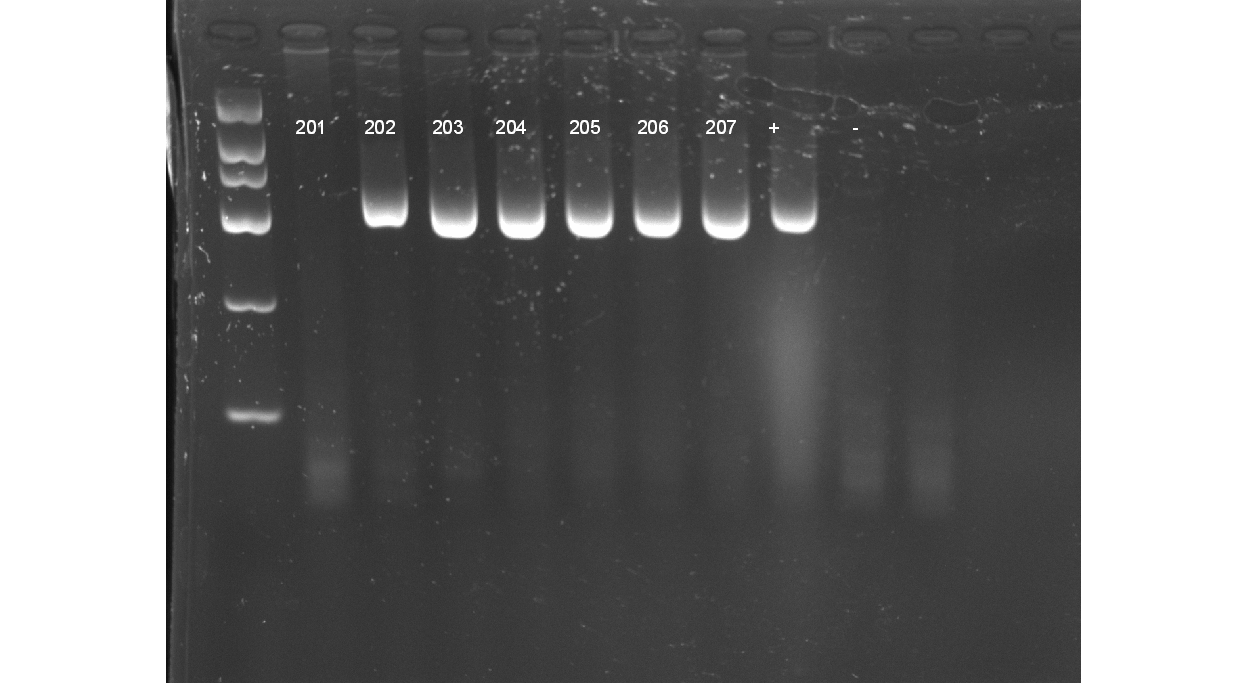
**

**
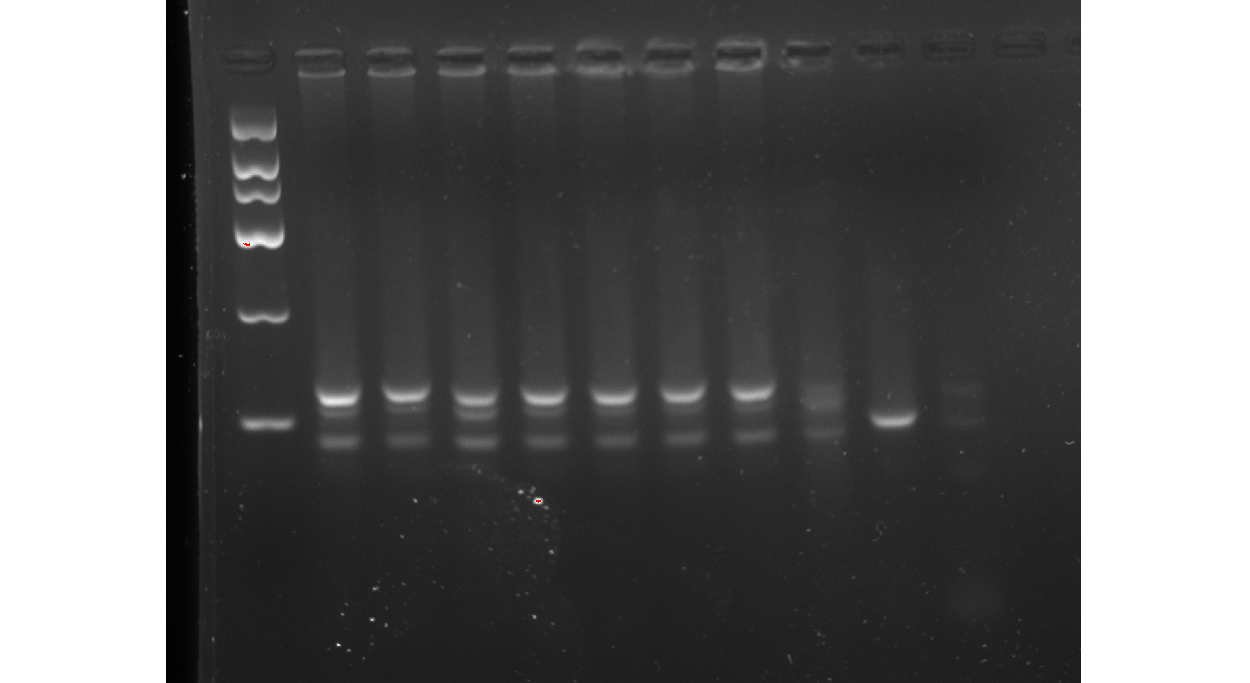
**

**Supplement figure 3 Original Figure of Figure 5A**


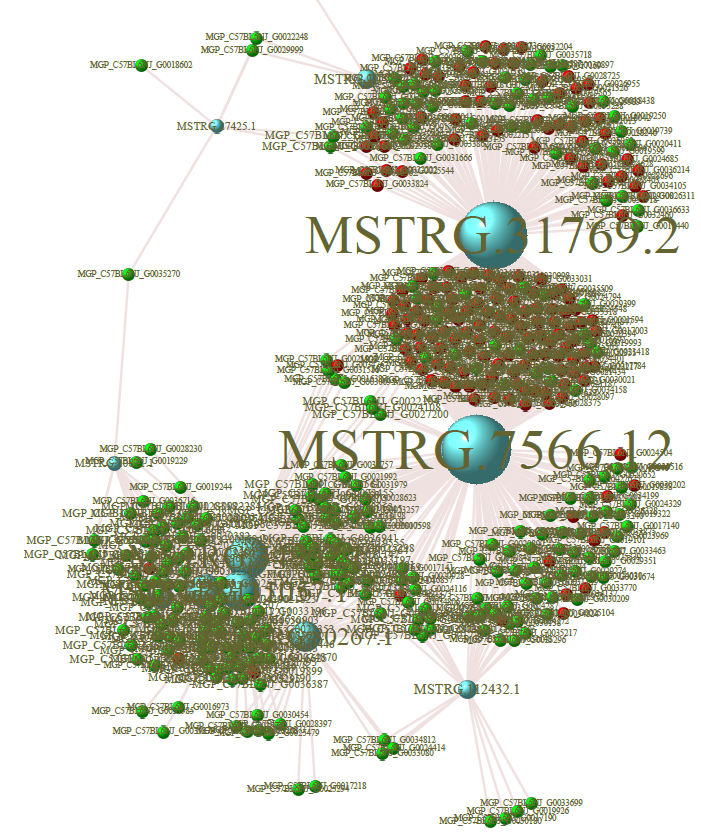


**Supplement figure 4 Original Figure of Figure 5C**

**
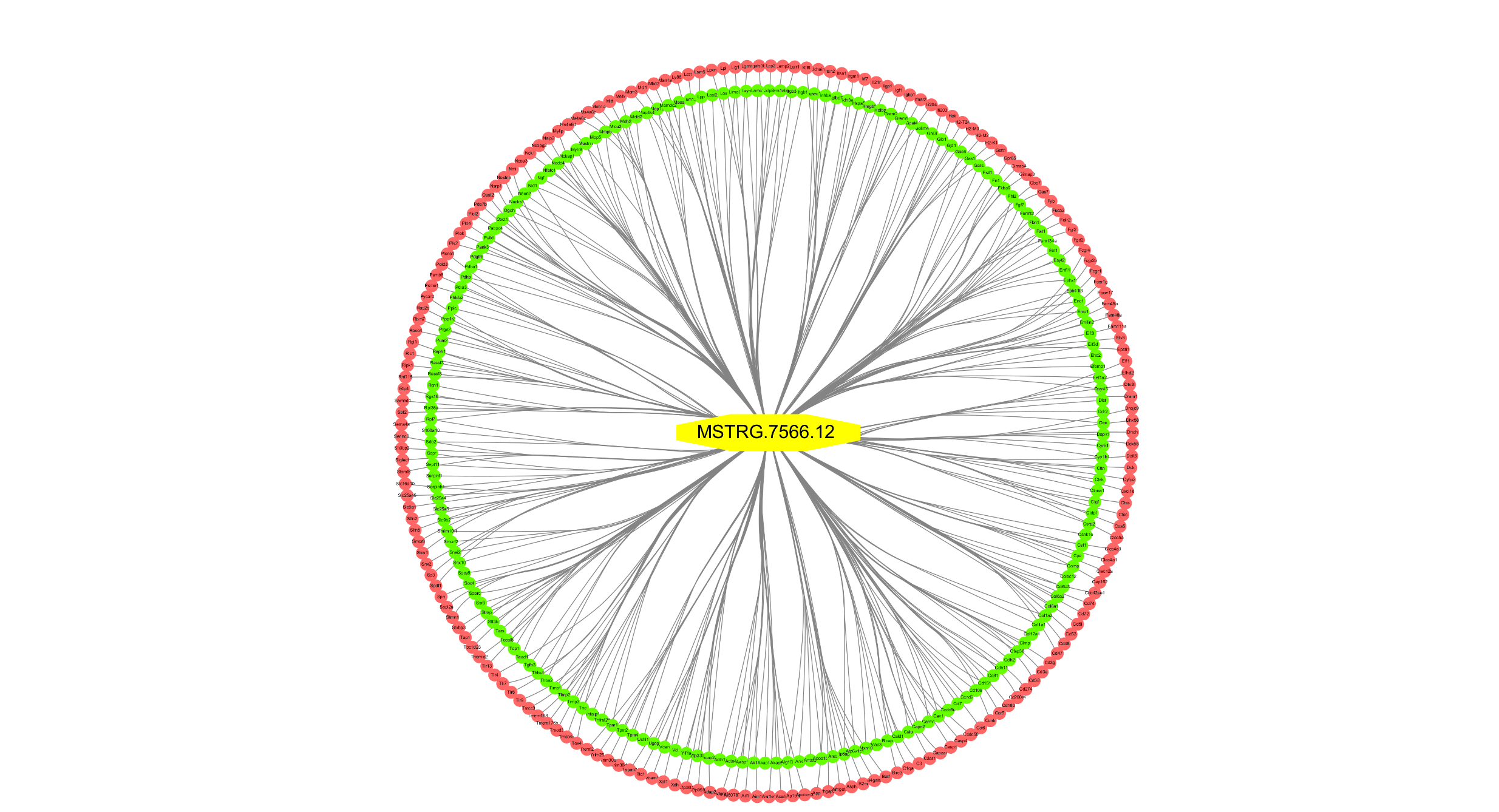
**

**Supplement figure 5 Original Figure of Figure 5F**

**
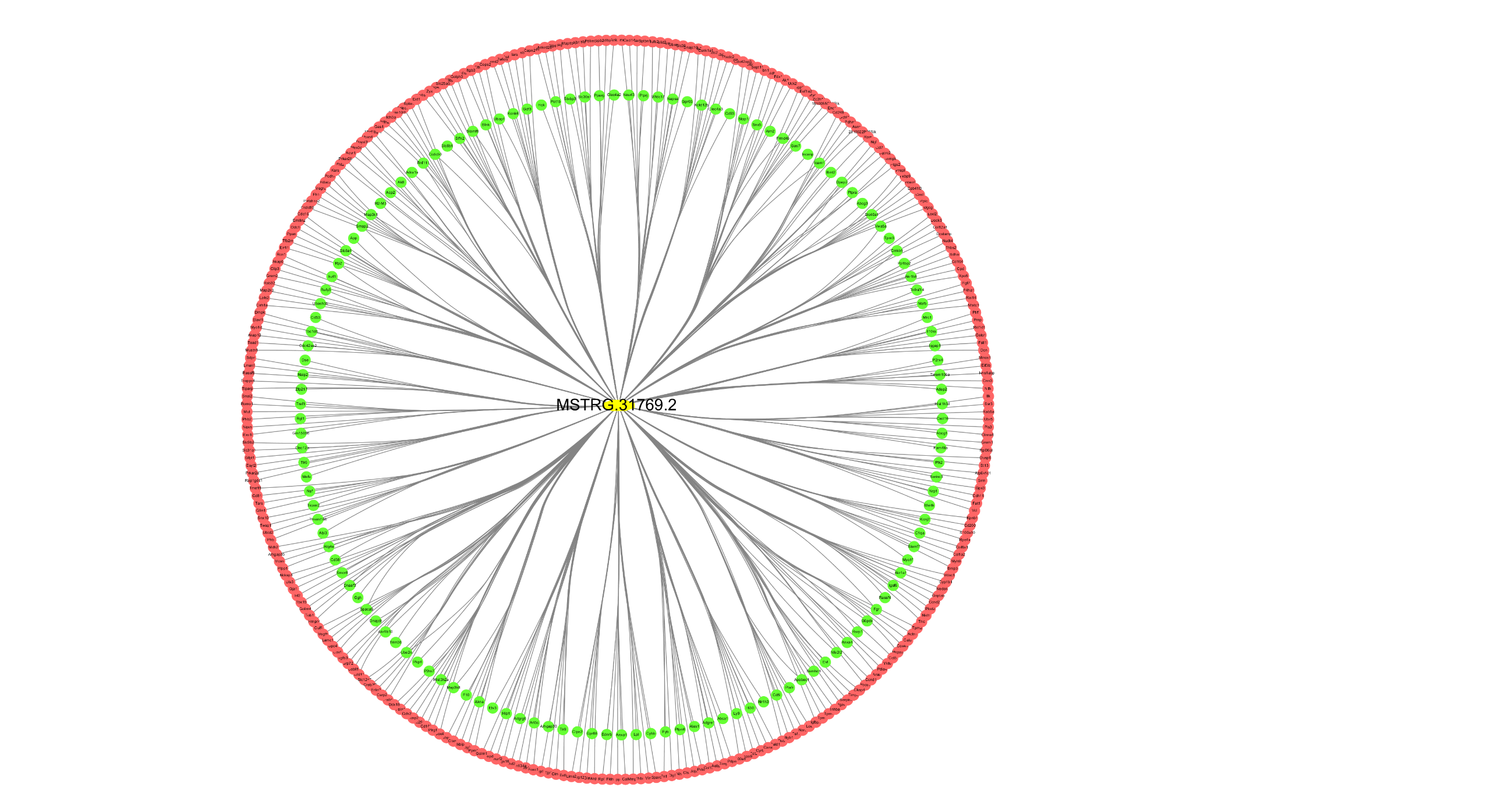
**
